# Identification of peripheral blood test parameters predicting the response to palbociclib and endocrine therapy for metastatic breast cancer: a retrospective study in a single institution

**DOI:** 10.1007/s00595-024-02893-z

**Published:** 2024-07-04

**Authors:** Misato Yamamoto, Masahiro Shibata, Aya Tanaka, Nobuyuki Tsunoda, Norikazu Masuda

**Affiliations:** 1 Department of Breast and Endocrine Surgery, Nagoya University Graduate School of Medicine, 65 Tsurumai-Cho, Showa-Ku, Nagoya, Aichi 466-8550 Japan; 2 Depatment of Surgery, Nagoya Ekisaikai Hospital, 4-66 Shounen-Cho, Nakagawa-Ku, Nagoya, Aichi 454-8502 Japan; 3 Department of Surgery, Inazawa Municipal Hospital, 100 Numa, Nazuka-Cho, Inazawa, Aichi 492-8510 Japan; 4 Department of Surgery,, Japanese Red Cross Aichi Medical Center Nagoya Daiichi Hospital, 3-35 Michishita-Cho, Nakamura-Ku, Nagoya, Aichi 453-8511 Japan

**Keywords:** Breast cancer, Palbociclib, C-reactive protein, Lactate dehydrogenase, Albumin

## Abstract

**Purpose:**

Cyclin-dependent kinase 4/6 inhibitors have been used in endocrine therapy for patients with estrogen receptor (ER)-positive and human epidermal growth factor receptor 2 (HER2)-negative metastatic breast cancer. Although randomized trials have shown that combined therapies prolong progression-free survival (PFS) in comparison to endocrine monotherapy, the predictors of efficacy are unknown. This study aimed to identify the blood test parameters to predict the effects of palbociclib and endocrine therapy.

**Methods:**

Seventy-nine patients treated with palbociclib and endocrine therapy between December 2017 and June 2022 were reviewed. We assessed PFS in patients according to factors evaluated based on patient characteristics and peripheral blood tests.

**Results:**

Patients in the C-reactive protein (CRP)-high, lactate dehydrogenase (LDH)-high, and albumin (Alb)-low groups had significantly shorter PFS than those in the normal group. A multivariate analysis revealed that high LDH and low Alb levels were independent factors that affected PFS. The Alb-low group had an inferior disease control rate. Patients in the CRP-high, LDH-high, and Alb-low groups who received these therapies as first- or second-line treatments showed poor PFS.

**Conclusions:**

Several predictors of the efficacy of palbociclib and endocrine therapy were identified in the peripheral blood test parameters of patients with ER-positive and HER2-negative subtypes of metastatic breast cancer.

## Introduction

Breast cancer is the most common malignancy in women [1]. It is categorized based on the expression of estrogen receptor (ER) and human epidermal growth factor 2 (HER2), and treatment strategies differ depending on the subtype. The most common ER-positive and HER2-negative subtypes are treated with tamoxifen or aromatase inhibitors after surgical intervention, which reduces the risk of recurrence [2]. Although various endocrine therapies and chemotherapeutic drugs are available for metastatic patients [3], a definitive cure remains elusive.

Cyclin-dependent kinase (CDK) 4/6 inhibitors such as palbociclib and abemaciclib have been used in combination with endocrine therapy in patients with ER-positive and HER2-negative metastatic breast cancer [3]. Although randomized trials have shown that these combined therapies prolong progression-free survival (PFS) relative to endocrine monotherapy [4, 5], some patients experience disease progression in a short period. Therefore, developing a method to predict the duration of the therapeutic effect of these drugs is crucial for providing optimal treatment for each patient.

Several recent studies have utilized peripheral blood tests for predicting drug efficacy, due to their ease and non-invasiveness [6, 7]. For example, a low neutrophil-to-lymphocyte ratio (NLR) and lymphocyte count are significantly associated with short PFS in metastatic breast cancer patients treated with eribulin mesylate [8, 9]. A study that assessed the effect of palbociclib, the first CDK4/6 inhibitor available in Japan, demonstrated that NLR and lymphocyte count can be predictive parameters of PFS [10]. However, that study also included patients treated with abemaciclib and ribociclib, in addition to patients treated with palbociclib. Furthermore, other parameters that can be investigated using blood tests have not been evaluated, including C-reactive protein (CRP), lactate dehydrogenase (LDH), and albumin (Alb), which have been reported as prognostic predictors not only in breast cancer [11–13], but also in other types of cancer [14, 15].

In this study, we aimed to identify parameters readily available in peripheral blood tests that could predict the effects of palbociclib and endocrine therapy in patients with ER-positive and HER2-negative metastatic breast cancer.

## Methods

### Patients and study design

In this single-center study, the clinical data of each patient were retrospectively analyzed. All female patients with ER-positive and HER2-negative metastatic breast cancer treated with palbociclib and endocrine therapies at Nagoya University Hospital between December 2017 and June 2022 were enrolled in this study. All patients were diagnosed with breast cancer based on pathological examination of the primary tumor. The expression of ER, progesterone receptor (PgR), and HER2 was examined by immunohistochemical staining of the primary tumor. A positivity rate of 1% or higher was considered positive for ER and PgR. The HER2 status was determined using immunohistochemical staining, with further fluorescence in situ hybridization tests conducted in HER2 2 + cases. ER and HER2 statuses were assessed in metastatic tissues if sampled. Metastases were diagnosed using imaging techniques, such as chest and abdominopelvic computed tomography (CT), bone scans, and positron emission tomography. Visceral metastases included any of the following baseline diseases: adrenal, bladder, central nervous system, esophagus, liver, lung, peritoneum, pleura, renal, small bowel, stomach, pancreas, thyroid, colon, rectum, ovary, biliary tract, ascites, pericardial effusion, spleen, or pleural effusion [16].

The patient was treated with palbociclib and endocrine therapies. Palbociclib was administered orally at an initial dose of 125 mg/day for 3 weeks on and 1 week off. The dose was then adjusted to either 100 mg or 75 mg based on the degree of side effects experienced by each patient. Endocrine therapy combined with palbociclib, aromatase inhibitors, or fulvestrant was selected at the physician’s discretion based on the treatment history for each patient. Aromatase inhibitors, such as anastrozole or letrozole, were administered orally every day, and fulvestrant was administered intramuscularly on days 1, 15, and 29, and thereafter once every 4 weeks.

### Peripheral blood tests

The following baseline data were collected from peripheral blood tests, which were performed immediately before palbociclib administration: white blood cell, neutrophil, lymphocyte, CRP, LDH, and albumin levels. The cutoff values of CRP, LDH, and Alb complied with the reference intervals for major clinical laboratory tests published by the Japanese Committee for Clinical Laboratory Standards [17], which was adopted by Nagoya University Hospital. The cutoff values of the lymphocyte count and NLR were 1500/µl and 3, respectively, according to previous studies [8, 9]. The NLR was calculated by dividing the number of neutrophils by the number of lymphocytes. The normal neutrophil level (3000/µl) was used as the cutoff value.

### Statistical methods

PFS was defined as the duration of palbociclib administration and endocrine therapy from the first day of treatment until the detection of disease progression. The treatment response was assessed using CT according to the new Response Evaluation Criteria in Solid Tumors (version 1.1) [18], laboratory data, and tumor markers. The disease control rate (DCR) was defined as the sum of complete response, partial response, and stable disease rates. Side effects were determined according to the Common Terminology Criteria for Adverse Events (version 5.0) [19]. PFS was examined using the log-rank test and Kaplan–Meier curves. The Cox proportional hazards model was used for univariate and multivariate analyses. The chi-square test was used to compare DCR and side effects. Correlations between the values of CRP, LDH, and Alb were analyzed using Spearman’s rank correlation test. All statistical analyses were performed using JMP16 (SAS Institute, Inc., Cary, NC, USA), and *P*-values of less than 0.05 (*P* < 0.05) were considered statistically significant.

## Results

### Patient characteristics

In total, 79 patients with ER-positive and HER2-negative metastatic breast cancer treated with palbociclib and endocrine therapies were evaluated. The patient characteristics are summarized in Table 1. The median patient age was 67 years (range, 38–88 years). The numbers of patients with de novo and recurrent tumors were 17 (21.5%) and 62 (78.5%), respectively. Immunostaining of PgR was positive (*n* = 69, 87.3%), negative (*n* = 8, 10.1%), or unknown (*n* = 2, 2.6%). There were 25 (31.6%) patients with visceral metastasis and 54 (68.4%) patients without visceral metastasis. The median number of treatment lines for metastatic breast cancer was 2 (range, 1–11). Combined endocrine therapy included aromatase inhibitors in 30 patients (38.0%) and fulvestrant in 49 patients (62.0%).

**Table 1:** Patient characteristics and clinical response

	Value
Age, median range (years)	67 (38–88)
de novo or recurrent tumor, *n* (%)	
de novo	17 (21.5%)
Recurrent	62 (78.5%)
*PgR* *status*, *n* (%)	
Positive	69 (87.3%)
Negative	8 (10.1%)
Unknown	2 (2.6%)
*Combined endocrine therapy*, *n* (%)	
Aromatase inhibitor	30 (38.0%)
Fulvestrant	49 (62.0%)
*Treatment lines for metastatic breast cancer*, *n* (%)	
1st	29 (36.7%)
2nd	19 (24.0%)
3rd and higher	31 (39.3%)
*Metastatic site*, *n* (%)	
Visceral	25 (31.6%)
Non-visceral	54 (68.4%)
*Clinical response*, *n* (%)	
CR	1 (1.2%)
PR	9 (11.4%)
SD	47 (59.5%)
PD	13 (16.5%)
Unknown	9 (11.4%)

*PgR* progesterone receptor, *CR* complete response, *PR* partial response, *SD* stable disease, *PD* progressive disease

### PFS outcome

**Fig. 1:**
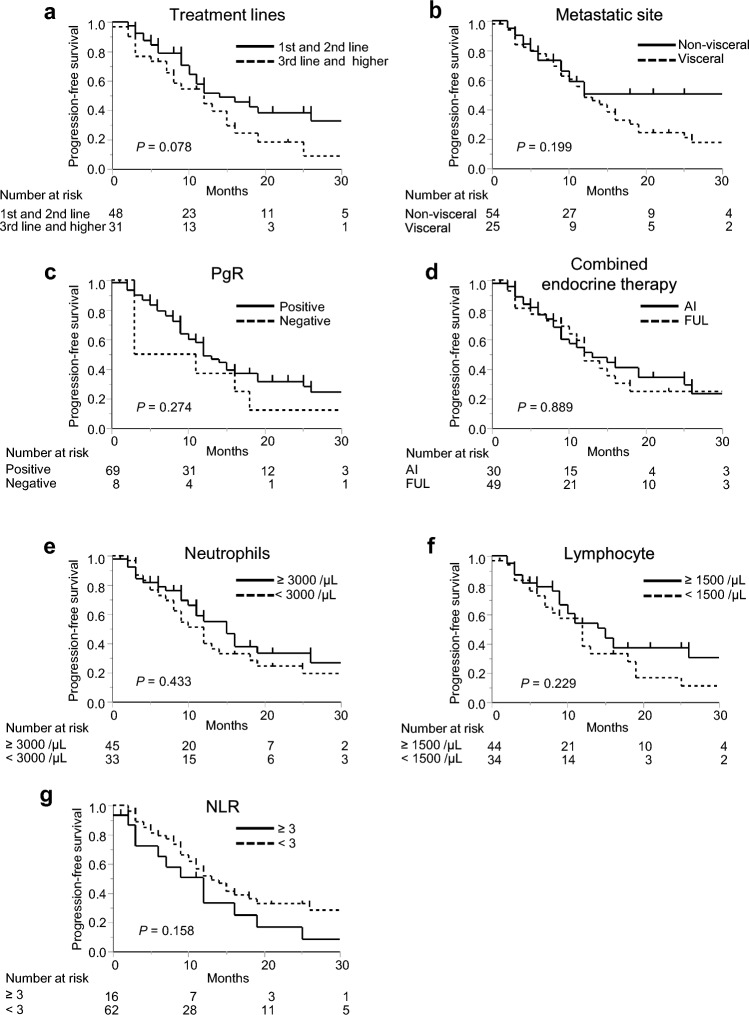
Kaplan–Meier curves for progression-free-survival (PFS). No significant differences were observed in PFS stratified according to the treatment lines (**a**), presence of visceral metastasis (**b**), progesterone receptor (PgR) positivity (**c**), type of combined endocrine therapy (**d**), baseline neutrophil count (**e**), lymphocyte count (**f**), or neutrophil-to-lymphocyte ratio (NLR) (**g**). AI, aromatase inhibitor; FUL, fulvestrant; and PgR, progesterone receptor

No significant differences were observed when PFS was stratified according to the treatment line (*P* = 0.078; Fig. 1a), presence of visceral metastasis (*P* = 0.199; Fig. 1b), PgR status (*P* = 0.274; Fig. 1c), or type of combined endocrine therapy (*P* = 0.889; Fig. 1d). We subsequently analyzed the parameters in the peripheral blood tests. PFS did not differ to a statistically significant extent when compared according to the neutrophil count (≥ 3000, *n* = 45, vs. < 3000, *n* = 33; *P* = 0.433; Fig. 1e), lymphocyte count (≥ 1500, *n* = 44, vs. < 1500, *n* = 34; *P* = 0.229; Fig. 1f), or NLR (≥ 3, *n* = 16, vs. < 3, *n* = 62; *P* = 0.158; Fig. 1g).

**Fig. 2:**
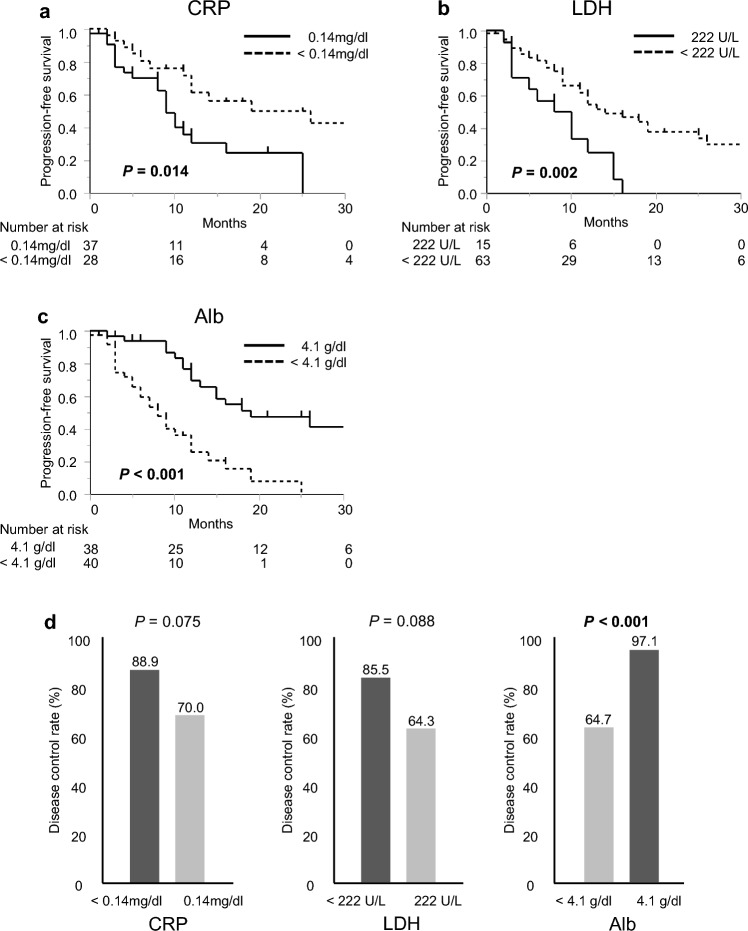
Progression-free-survival (PFS) curves in patients stratified according to the factors evaluated by peripheral blood tests. PFS was significantly shorter in the C-reactive protein (CRP)-high (**a**), lactate dehydrogenase (LDH)-high (**b**), and albumin (Alb)-low (**c**) groups than in the normal group. The CRP-high and LDH-high groups tended to show an inferior disease control rate (DCR) relative to the CRP-normal and LDH-normal groups, respectively. The Alb-low group experienced inferior DCR in comparison to the Alb-normal group (**d**)

Following the evaluation of patient biochemical factors, namely CRP, LDH, and Alb, the patients were grouped based on these parameters using the standard values adopted for these factors in the facility. Patients with CRP ≥ 0.14 mg/dl and CRP < 0.14 mg/dl were classified into the CRP-high (*n* = 37) and CRP-normal (*n* = 28) groups, respectively. Patients with LDH ≥ 222 U/L and LDH < 222 U/L were classified into the LDH-high (*n* = 15) and LDH-normal (*n* = 63) groups, respectively. Patients with Alb < 4.1 g/dl and Alb ≥ 4.1 g/dl were classified into the Alb-low (*n* = 40) and Alb-normal (*n* = 38) groups, respectively. Patients in the CRP-high, LDH-high, and Alb-low groups showed significantly shorter PFS than those in the CRP-normal (*P* = 0.014; Fig. 2a), LDH-normal (*P* = 0.002; Fig. 2b), and Alb-normal groups (*P* < 0.001; Fig. 2c).

The clinical responses of the patients were classified as follows: complete response, *n* = 1 (1.2%); partial response, *n* = 9 (11.4%); stable disease, *n* = 47 (59.5%); and progressive disease, *n* = 13 (16.5%). The clinical response was unknown in 9 patients because of discontinuation of the treatment due to side effects or because the patient moved before evaluation (Table 1). When the DCR was compared between the CRP, LDH, and Alb groups, the CRP-high and LDH-high groups tended to show inferior DCR in comparison to the CRP-normal and LDH-normal groups, respectively (CRP, 70% vs. 88.9%; *P* = 0.075; LDH, 64.3% vs. 85.5%; *P* = 0.088). The Alb-low group showed inferior DCR in comparison to the Alb-normal group (64.7% vs. 97.1%; *P* < 0.001; Fig. 2d).

### Identification of predictive factors of PFS

The univariate analyses for each clinical and biological factor that predicted PFS showed that PgR-negative status (*P* = 0.285), visceral metastasis (*P* = 0.213), number of regimens (≥ 3; *P* = 0.088), type of endocrine therapy (fulvestrant; *P* = 0.891), lymphocyte count (< 1500; *P* = 0.242), and NLR (≥ 3; *P* = 0.172) were not significant predictive factors. Meanwhile, CRP-high (hazard ratio: 2.46; 95% confidence interval (CI) 1.15–5.23; *P* = 0.019), LDH-high (hazard ratio: 2.75; 95% CI 1.39–5.31; *P* = 0.003) and Alb-low (hazard ratio: 3.92; 95% CI 2.02–7.59; *P* < 0.001) statuses were statistically significant predictive factors. A subsequent multivariable analysis identified LDH-high (hazard ratio: 3.19; 95% CI 1.23–8.25; *P* = 0.017) and Alb-low (hazard ratio: 3.23; 95% CI 1.15–9.09; *P* = 0.026) statuses as independent predictors of poor PFS (Table 2).

**Table 2:** Univariate and multivariate analysis for factors predicting progression-free survival

		Univariate			Multivariate		
Variables	*n*	Hazard ratio	95% CI	*P*-value*	Hazard ratio	95% CI	*P*-value*
PgR (negative)	8	1.55	0.69–3.50	0.285	2.28	0.71–7.32	0.163
Visceral	25	1.62	0.76–3.51	0.213	1.35	0.50–3.29	0.504
Treatment lines (3rd and higher)	31	1.68	0.92–3.06	0.088	1.66	0.63–4.36	0.301
Endocrine therapy (FUL)	49	1.04	0.57–1.92	0.891	1.15	0.52–2.54	0.726
Lymphocyte (< 1500/μL)	34	1.42	0.79–2.58	0.242	1.56	0.58–4.22	0.377
NLR (≥ 3)	16	1.58	0.32–1.22	0.172	1.25	0.44–3.57	0.668
CRP (≥ 0.14 mg/dL)	37	2.46	1.15–5.23	0.019*	1.56	0.57–4.22	0.384
LDH (≥ 222 U/L)	15	2.75	1.39–5.31	0.003*	3.19	1.23–8.25	0.017*
Alb (< 4.1 g/dL)	40	3.92	2.02–7.59	< 0.001*	3.23	1.15–9.09	0.026*

*CI* confidence interval, *PgR* progesterone receptor, *FUL* fulvestrant, *NLR* neutrophil-to-lymphocyte ratio, *CRP* C-reactive protein, *LDH* lactate dehydrogenase, *Alb* albumin, *P < 0.05

Next, the correlations among the CRP, LDH, and Alb values that affected PFS in the univariate analysis were evaluated. The CRP level was negatively correlated with the Alb level (correlation coefficient,  −  0.431; *P* < 0.001), whereas no correlation was observed between the CRP and LDH levels (correlation coefficient, 0.069; *P* = 0.587) or LDH and Alb levels (correlation coefficient, 0.021; *P* = 0.854; Table 3).

**Table 3:** Correlation between C-reactive protein, lactate dehydrogenase, and albumin levels

	The Spearman rank-order correlation coefficients	*P*-value*
CRP vs. LDH	0.069	0.587
CRP vs. Alb	− 0.431	< 0.001*
LDH vs. Alb	0.021	0.854

*CRP* C-reactive protein, *LDH* lactate dehydrogenase, *Alb* albumin, ^*^P < 0.05

### PFS in patients who received palbociclib combined with endocrine therapy as the 1st or 2nd line treatment

**Fig. 3:**
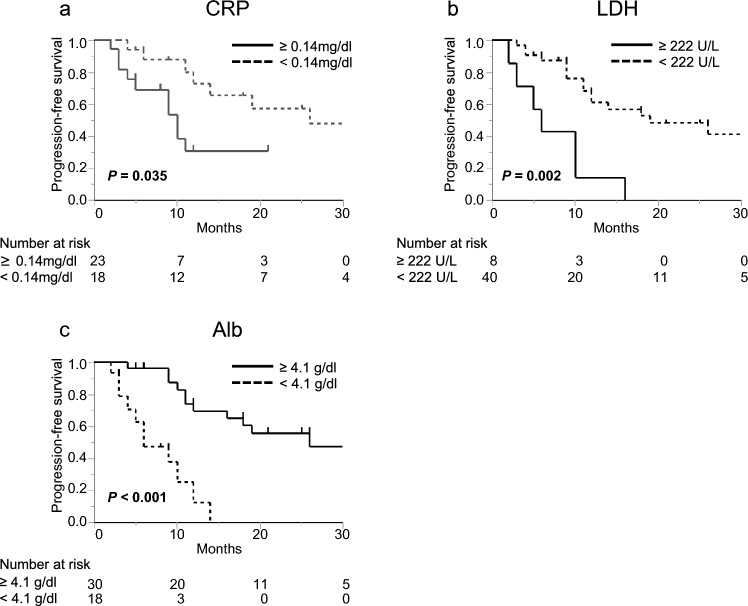
Progression-free-survival (PFS) curves in patients who received palbociclib and endocrine therapy as first- or second-line treatment. Patients were stratified according to the standard values of C-reactive protein (CRP) (**a**), lactate dehydrogenase (LDH) (**b**), or albumin (Alb) (**c**). Patients with high CRP, high LDH, or low Alb levels experienced shorter PFS than those with normal CRP, LDH, or Alb levels, respectively

Since palbociclib combined with endocrine therapy has been primarily used as first- or second-line treatment [4, 5], we focused on 48 patients who received this therapy as the first- or second-line treatment. The CRP-high (*n* = 23), LDH-high (*n* = 8), and Alb-low (*n* = 18) groups had shorter PFS than the CRP-normal (n = 18; *P* = 0.035; Fig. 3a), LDH-normal (*n* = 40; *P* = 0.002; Fig. 3b), and Alb-normal groups (*n* = 30; *P* < 0.001; Fig. 3c).

### Association between drug side effects and CRP, LDH, and Alb levels

Severe side effects that caused the discontinuation and reduced the duration of palbociclib and endocrine therapies were fatigue (Grade ≥ 3), decreased neutrophil counts (Grade ≥ 3), increased aspartate aminotransferase (AST) or alanine aminotransferase (ALT) levels (Grade ≥ 3), and pneumonitis (Grade ≥ 2). The CRP-normal group more frequently showed a reduced neutrophil count in comparison to the CRP-high group (*P* < 0.001; Table 4). The mean baseline neutrophil count in the CRP-normal group was lower than that in the CRP-high group (mean ± standard deviation, 2868 ± 737 vs. 3762 ± 1477; *P* = 0.005). Furthermore, the CRP level was not associated with the frequency of other severe side effects (fatigue, *P* = 0.724; increased AST or ALT, *P* = 0.192; pneumonitis, *P* = 0.129). The LDH and Alb levels and the frequency of severe side effects in the groups did not differ to a statistically significant extent.

**Table 4:** Association between the frequency of side effects causing discontinuation and reduction of palbociclib and C-reactive protein, lactate dehydrogenase, and albumin levels

	CRP			LDH			Alb		
	Normal < 0.14 mg/dl *n* (%)	High ≥ 0.14 mg/dl *n* (%)	*P*-value	Normal < 222U/L *n* (%)	High ≥ 222U/L *n* (%)	*P*-value	Low < 4.1 g/dl *n* (%)	Normal ≥ 4.1 g/dl *n* (%)	*P*-value
Fatigue (Grade ≥ 3)	1 (1.5)	2 (3.1)	0.724	3 (3.9)	0 (0.0)	0.253	3 (3.9)	0 (0.0)	0.085
AST or ALT increased (Grade ≥ 3)	1 (3.6)	0 (0.0)	0.192	2 (3.2)	0 (0.0)	0.352	0 (0.0)	2 (5.3)	0.087
Neutrophil count decreased (Grade ≥ 3)	25 (89.3)	19 (51.4)	< 0.001*	45 (71.4)	10 (66.7)	0.717	26 (65.0)	29 (76.3)	0.272
Pneumonitis (Grade ≥ 2)	0 (0.0)	2 (5.4)	0.129	2 (3.2)	0 (0.0)	0.352	1 (2.5)	1 (2.6)	0.971

*CRP* C-reactive protein, *LDH* lactate dehydrogenase, *Alb* albumin, *AST* aspartate aminotransferase, *ALT* alanine aminotransferase, *P < 0.05

## Discussion

This study demonstrated the utility of CRP, LDH, and Alb as predictive factors for the response to palbociclib and endocrine therapy in patients with ER-positive and HER2-negative subtypes of metastatic breast cancer. Among them, high LDH and low Alb levels were independent predictors of short PFS in the multivariate analysis.

Recent studies have focused on the use of peripheral blood data for predicting the prognosis and drug effects because peripheral blood collection can be readily performed with minimal invasiveness [6, 7]. In breast cancer patients treated with eribulin mesylate, the NLR is reportedly associated with PFS, which is significantly shorter at NLR > 3 [8, 9]. However, studies evaluating the predictive factors for the efficacy of palbociclib and endocrine therapies are limited. Roitter et al. [10] examined the PFS of patients treated with CDK4/6 inhibitors and endocrine therapy as first-line treatment and demonstrated that PFS was shorter in patients with an NLR > 2.53. This study included several CDK4/6 inhibitors (e.g., palbociclib, ribociclib, and abemaciclib); however, biochemical data were not examined.

In the current study, PFS was not significantly associated with treatment line, presence of visceral metastases, PgR expression, or combined endocrine therapy. Notably, patients with CRP-high, LDH-high, or Alb-low disease had a poor PFS. The association with the NLR, which was reported in a previous study [10], was not recognized as a significant factor. This is possibly because previous studies have included other CDK4/6 inhibitors. Although CRP, LDH, and Alb were found to be factors that affected PFS in the univariate analysis, in the multivariate analysis, only LDH and Alb were identified as independent factors. Further results showed a significant negative correlation between CRP and Alb levels, suggesting that the CRP level was influenced by the Alb level in these patients. This was consistent with previous reports showing that CRP and Alb were negatively correlated in patients with cancer [20, 21]. Although LDH, CRP, and Alb levels have been reported as prognostic factors in advanced recurrent breast cancer [11–13], these previous studies included patients with a poor nutritional status. In contrast, in our study, patients were categorized by institutional reference values and included patients who were still able to receive medication. Therefore, these factors are considered useful novel predictors of both the prognosis and therapeutic efficacy.

As these results are based on observational data, explaining how LDH, CRP, and Alb levels affect PFS is difficult. Nevertheless, based on previous studies, these values seem to reflect the molecular status of hypoxia and inflammation, which leads to resistance to palbociclib and endocrine therapy [22]. Increased LDH levels are associated with a poor prognosis in various cancers [11]. When PD-1 inhibitory drugs are used to treat patients with non-small cell lung cancer and melanoma, patients with high LDH show a poor prognosis [14, 23] because elevated LDH induces cytokine secretion from cancer cells involved in inflammation [14]. In breast cancer, vascular endothelial growth factor (VEGF) and hypoxia inducible factor (HIF)-1 are elevated in hypoxic environments, which attenuates the effects of CDK4/6 inhibitors [22, 24, 25]. A positive association between the levels of LDH, VEGF, and HIF-1 has been shown not only in breast cancer but also in gastric cancer [25, 26]. Thus, elevated LDH levels indicate a hypoxic tumor environment which may lead to poorer treatment outcomes. Another resistance mechanism to palbociclib and endocrine therapy is the inactivation of retinoblastoma protein (Rb) due to hyperphosphorylation [27, 28]. Interleukin-6 (IL-6), which mediates Rb phosphorylation, is elevated in cancer patients with high CRP and low Alb levels [20, 29–31]. Thus, high CRP and low Alb levels reflect elevated IL-6 levels, leading to RB inactivation.

In the current study, we included patients who started treatment with palbociclib and endocrine therapy as the third or higher line of treatment. This was because we wanted to capture patients who started treatment when palbociclib was launched. Palbociclib and endocrine therapy are currently recommended for use as first- or second-line therapy; hence, we evaluated 48 patients who were treated with palbociclib as the first- or second-line treatment, which showed robustness in the utility of CRP, LDH, and Alb levels as predictive factors.

As we speculated that the frequency of side effects affected the duration of PFS, we further evaluated whether the levels of CRP, LDH, and Alb influenced the frequency of side effects. We found that the frequency of neutropenia was high in the CRP-normal group, whereas no other associations were observed between the frequency of other side effects and CRP, LDH, or Alb levels. This may be because the baseline neutrophil count was lower in the CRP-normal group. Despite lower baseline neutrophil counts, the PFS was longer in the CRP-normal group. In addition, the reduction in palbociclib due to neutropenia did not affect the therapeutic effect in randomized prospective clinical trials [5]. Based on these findings, the significant associations between PFS and CRP, LDH, and Alb levels were not considered because of the frequency of side effects leading to dose reduction of palbociclib.

These results may be applicable to clinical practice. That is, when a patient’s PFS is expected to be short based on the results of blood tests before the initiation of treatment, frequent imaging tests can be recommended. This would enable physicians to quickly switch to better therapeutic options in the event of disease progression.

The present retrospective study was associated with several limitations. First, several values were missing from the dataset. Second, the methods used to determine therapeutic effects varied among patients. Although imaging tests were performed every 3 or 4 months in most cases, a few cases were judged based on trends in tumor markers and clinical symptoms. Hence, these results should be prospectively validated in a cohort study.

In conclusion, this study identified several factors predicting the efficacy of palbociclib and endocrine therapy using a simple and minimally invasive peripheral blood test. We believe that these factors would be useful for individually determining whether combined palbociclib and endocrine therapy is the appropriate course of action for each patient.
